# Practical Notes and Formulæ

**Published:** 1881-01-20

**Authors:** 


### PRACTICAL NOTES AND FORMULÆ.

**Warner's Parvules.**—We have before alluded to the excellency, purity and practical convenience of these unique and beautiful preparations, manufactured by Wm. R. Warner & Co., Philadelphia. We carry them in our pocket-case daily and find little use for the apothecary, as we can readily administer the different ingredients in any required fraction or quantity by giving one or more of the different parvules, so as to meet the indications of the case.

We find them very efficient, safe and reliable. Children will usually swallow them without difficulty, and they are borne by the most delicate stomachs in cases that will not tolerate the usual methods of administering drugs. We seldom fail to induce children to take the quinine parvules, who ordinarily cannot be persuaded to take the remedy in powder or solution, or if forced down they will at once eject it by vomiting.

**Treatment of Diphtheria by Tartaric Acid.**—M. Vidal advocates the employment of tartaric acid in diphtheria (*La France Medicale*). Local action on the false membrane is necessary because they have a great tendency to propagation by a sort of auto-inoculation comparable to that which takes place in certain skin diseases. The formula he employs is this:

| | |
|---|---|
| Tartaric acid,........................................ | 10 grammes. |
| Glycerine,........................................ | 15 grammes. |
| Distilled mint water........................................ | 25 grammes. |

The tartaric acid acts on the false membrane, which it changes into a gelatinous mass and favors its expulsion. Applications of it should be made about every three hours, and should be followed a short time after by applications of lemon juice.—*Cincinnati Lancet and Clinic.*

**Petroleum Mass.**—Dr. Milton, in Maryland Medical Journal, says: The following preparation of petroleum has afforded me more gratifying results in chronic bronchitis, laryngitis and obscure pains of the lungs, than anything else. The formula recommended by Dr. M. Griffith, of Irving, N. Y., is as follows:

| | | |
|---|---|---|
| ℞ | Petroleum mass,........................................ | ℥ j. |
| | Pulv. cubebæ,........................................ | aa ℥ ss. |
| | Pulv. Doveri,........................................ | |

Make 4 grain pills. Sig.—One pill every 3 or 4 hours.

Chlorate of potash to be used in making Dover's powders instead of sulphate of potash.

**Ergotin Solution for Hypodermic Use.**—

| | | |
|---|---|---|
| ℞ | Ergotini........................................ | gr. 36 |
| | Glycerinæ........................................ | |
| | Aquæ........................................ | aa ♏ 108 |

Mix.

**Removal of Spots, Stains, Etc.**—The following are extracted from a German journal:

*Matter Adhering Mechanically.*—Beating, bruising and currents of water, either on the upper or under side.

*Gum, Sugar, Jelly, etc.*—Simply washing with water at a hand heat.

*Grease.*—White goods, wash with soap or alkaline lyes. Colored cottons, wash with French chalk or fuller's earth, and dissolve away with benzin or ether.

*Oil Colors, Varnish and Resins.*—On white or colored linens, cottons or woolens, use rectified oil of turpentine, alcohol lye and their soap. On silks, use benzin, ether and mild soap, very cautiously.

*Stearin.*—In all cases, strong, pure alcohol.

*Vegetable Colors, Fruit, Red Wine and Red Ink.*—On white goods, sulphur fumes or chlorine water. Colored cottons and woolens, wash with lukewarm soap lye or ammonia. Silk the same, but more cautiously.

*Alizarian Inks.*—White goods, tartaric acid, the more concentrated the older are the spots. On colored cottons and woolens and on silks, dilute tartaric acid is applied cautiously.

*Blood and Albuminoid Matters.*—Steeping in lukewarm water. If pepsin or the juice of Carica papya can be procured, the spots are first softened with lukewarm water, and then either of these substances are applied.

*Iron Spots and Black Ink.*—White goods, hot oxalic acid, dilute muriatic acid, with little fragments of tin. On fast dyed cottons and woolens, citric acid is cautiously and repeatedly applied. Silks, impossible.

*Lime and Alkalies.*—White goods, simple washing. Colored cottons, woolens and silks are moistened, and very dilute citric acid is applied with the finger end.

*Acids, Vinegar, Sour Wine, Must, Sour Fruits.*—White goods, simple washing, followed up by chlorine water if a fruit color accompanies the acid. Colored cottons, woolens and silks are very carefully moistened with dilute ammonia, with the finger end. [In case of delicate colors, it will be found preferable to make some prepared chalk into a thin paste, with water, and apply it to the spots.]

*Tanning from Chestnuts, Green Walnuts, etc., or Leather.*—White goods, hot chlorine water and concentrated tartaric acid. Colored cottens, woolens and silks, apply dilute chlorine water cautiously to the spot, washing it away, and reapplying it several times.

*Tar, Cart Wheel Grease, Mixtures of Fat, Rosin and Acetic Acid.*—On white goods, soap and oil of turpentine, alternating with streams of water. Colored cottons and woolens, rub in with lard, let lie, soap, let lie again, and treat alternately with oil of turpentine and water. Silks the same, more carefully, using benzin instead of the oil of turpentine.

*Scorching.*—White goods, rub well with linen rags dipped in chlorine water. Colored cottons, redye if possible, or in woolen raise a fresh surface. Silks, no remedy.

**Cancer Cure.**—

| | | |
|---|---|---|
| ℞ | Arsenic........................................ | |
| | Rochell Salts........................................ | |
| | White vitriol........................................ | |
| | Sulphur........................................ | aa ʒ i |

Mix with yolk of eggs to the consistence of butter, and put into a new earthen dish; put into a brick or stone oven; slowly bake it until it rises up higher than the top of the dish, like a well done, rich cake, let it cool and rub up fine; mix a little of the above with the yolk of an egg and apply to the cancer.

Change every two days; do not make the plaster quite as large as the cancer, for it will find the whole of the cancer, let it extend ever so far. Salve to use after the cancer is out:

| | |
|---|---|
| Fresh butter or lard........................................ | ℔ i. |
| Beeswax, ........................................ | ℥ iv. |
| Pine turpentine,........................................ | ℥ vi. |
| Pure honey,........................................ | ℥ ij. |
| Resin,........................................ | ℥ iij. |

Melt all together, and set off the vessel from the stove, and partly cool; add half ounce of finely pulverized verdigris; stir until cool.

This salve is to be used first; afterwards the following to heal the sore:

| | |
|---|---|
| Hog's lard........................................ | ℥ iv. |
| Beeswax,........................................ | ℥ ivss. |

Melt together and stir until cool. Also for cancer a middle receipt:

| | |
|---|---|
| White vitriol,........................................ | 2 parts. |
| Arsenic,........................................ | 1 part. |
| Corrosive sublimate,........................................ | ½ part. |

Mix the powder with simple cerate. Mix well and apply a little of this to the cancer every day, until it is out, and heal it with the healing salve. The foregoing prescriptions enjoy great celebrity in certain parts of our State, and undoubted cures have been known to result from their use.

The practitioner will be able to decide from the nature of each case, the length of time these applications should be continued, etc.—*Med. Independent.*

**Amenorrhœa, Dysmenorrhœa, Etc.**—Dr. W. K. Bowling, of Nashville, Tenn., regards spirits of ammonia as specific in all the above diseases, in relieving the terrible sufferings of the patient. Use the following, viz: Put a drachm of spirits of ammonia in a chamber, and require the patient to sit on it a few moments. Dr. Bowling regards this a "Discovery" in medicine.

**McMunn's Elixir.**—In place of this nostrum there has been adopted into the U. S. Pharm. a formula for tinctura opii deodorata. This preparation, as furnished to the hospitals of the department, is assayed and adjusted to the strength of 4 grs. of morphia in 1 fl. ℥.—*Monthly Review of Medicine and Pharmacy.*

**Two Favorite Prescriptions.**—Prof. J. S. Wellford, M. D., reports the following prescription valuable in cases of lactation, general debility, anæmia, and all cases requiring a general nervine and tonic:

℞ Tr. ferri. chlorid,........................................ ʒ vi.

Acid Phosph. dil........................................ ʒ vi.

Quinidiæ sulph........................................ ℈ ii.

Strychniæ,........................................ gr. ½.

Syr. simpl........................................ ʒ iv.

Aq. destil,........................................ qs. ʒ iv.

M. ft. mist. S. One teaspoonful in water three times a day.

Dr. Wellford also uses the following with much success in constipation, either of pregnancy or of a general and habitual character:

℞ Pulv. fol. sennæ........................................ ʒ iij.

Sem. anisi........................................ ʒ i.

Rad. glycyrrhizæ,........................................ ʒ i.

Sulphur sublim........................................ ʒ i.

Sacchar alb........................................ ʒ iii.

M. ft. pulv. S. One teaspoonful stirred in a wineglassful of water, and taken at bed-time.—*Clinic.*

**Asthma.**—

℞ Chloral Hydrate........................................ ʒ v.
Potassii Bromidi,........................................ ʒ ijss.
Syrup Flores Aurantii Aquæ Distillat,.......... aaf ℥ i. M.
S. One teaspoonful in half a wine glassful of water every two hours until sleep is induced or dyspnœa is relieved.

Or, the following:

℞ Grindelia Robusta,........................................ ¾ ounce.
Yerba Santa........................................ ¼ ounce. M
Sig. One-half to one teaspoonful, more or less, according to age, every half, one or two hours; be governed by its effect on the disease.—*Ind. Pract.*

**Lent's Solution of Quinia.**—

℞ Quiniæ Sulphat........................................ gr. 80
Aquæ........................................ fl. ℥ 1
Acidi Sulphur. dil........................................ q. s.
Heat to boiling and add Acidi Carbolici............ gr. 5
For Hypodermic use.

**Burns.**—Dr. A. L. Barry, of Ringold, Ga., says, that a solution of alum in water forms an excellent application to burns, relieving the pain in a few minutes, and leading to a rapid healing of the inflamed surface.

℞ Alum........................................ 1 ounce.
Water........................................ 1 quart.

Sig. Keep a cloth wet with the solution constantly applied.

